# Genitourinary tuberculosis presenting as treatment resistant dysuria in a young patient: a case report

**DOI:** 10.1093/jscr/rjae818

**Published:** 2024-12-26

**Authors:** Louis Woodward, Ali Sahin, Stefanos Almpanis

**Affiliations:** Department of Urology, North Middlesex University Hospital, Sterling Way, London N18 1QX, United Kingdom; Department of Urology, North Middlesex University Hospital, Sterling Way, London N18 1QX, United Kingdom; Department of Urology, North Middlesex University Hospital, Sterling Way, London N18 1QX, United Kingdom

**Keywords:** tuberculosis, genitourinary, dysuria

## Abstract

Genitourinary tuberculosis is the second most common form of extrapulmonary tuberculosis. We present the case of a male patient in his late 20s who presented to his general practitioner with symptoms of recurrent urinary tract infection (UTI). Upon investigation his estimated glomerular filtration rate was found to be 61 ml/min/1.75 m^2^ and an ultrasound scan of the kidney, ureters, and bladder revealed a left sided hydronephrosis. A computerized tomography urogram confirmed upper and lower pole lesions of the left kidney with ureteric changes and lymphadenopathy consistent with chronic atypical infection. A urine acid-fast bacilli culture was positive for urinary tuberculosis (TB). The patient’s disseminated TB was treated with conventional anti-TB medications. Our case report highlights the value in considering genitourinary tuberculosis amongst the list of differential diagnoses in younger patients presenting with symptoms of recurrent UTI.

## Introduction

Tuberculosis (TB) is an infection caused by Mycobacterium tuberculosis and has an incidence of 7.8 per 100 000 in England [1]. The lungs are the most common site of infection, however, up to 40% of patients have extra-pulmonary manifestations and of those, 40% can involve the urogenital tract [2]. It has been noted that genitourinary TB (GUTB) is the second most common form of extra-pulmonary TB after lymph node [3]. GUTB can affect anywhere along the urogenital tract but most commonly affects the kidneys. Uniquely, GUTB can spread between individuals during sexual intercourse [2].

## Case report

A man in this late 20s was referred to urology by his general practitioner with recurrent dysuria and grade 2 hydronephrosis found on ultrasound scan of the kidney, ureters, and bladder. He was born in the UK with no known TB contacts but had spent several months in Bangladesh across his life. In clinic, he was found to have an estimated glomerular filtration rate of 61 ml/min/1.75 m^2^. A CT scan of the kidneys showed a lesion in the lower pole of the left kidney, left hydronephrosis, and urothelial thickening consistent with an infective or inflammatory aetiology*.* The following month, a CT-urogram confirmed upper and lower pole lesions of the left kidney with ureteric changes and lymphadenopathy consistent with chronic atypical infection*.* On account of the abnormal imaging, cystoscopy was performed revealing a markedly inflamed bladder with unidentifiable ureteric orifices. Histological analysis of three bladder biopsies taken during the procedure showed intensely inflamed and ulcerated mucosa. The underlying stroma contained sheets and ill-defined aggregates of macrophages, some of which were epithelioid in appearance. Following this, a urine AFB culture was requested and found to be positive for *M. tuberculosis*. Whole genome sequencing confirmed sensitivity to isoniazid, rifampicin, ethambutol, pyrazinamide, and quinolones. Sputum and cerebrospinal fluid AFB cultures were negative.

Following diagnosis of renal TB, the patient began to suffer from headaches with associated nausea. A magnetic resonance imaging (MRI) scan of the brain revealed a solitary right cerebellar tuberculoma and he was treated with rifater, ethambutol and pyridoxine plus dexamethasone to reduce cerebral swelling. He will have a repeat MRI to ensure resolution of the tuberculoma.

## Discussion

TB is the most common cause of infection related death globally, with an incidence in the UK of 7.8 per 100 000 [1, 2]. GUTB is defined as an infection of the genitals and/or the urinary tract by a bacterium of the *M. tuberculosis* complex [2]. Of cases of pulmonary TB in developed countries, 2–10% may go on to develop GUTB. Genitourinary infection may result from hematogenous spread from an initial pulmonary infection site or as a primary infection of the genitourinary tract through, for example sexual transmission [3, 4].

As was the case for our patient, the kidney is the most common site of infection and may produce symptoms including fever, weight loss, loin pain, haematuria, and voiding symptoms but this may vary by locus of infection along the urogenital tract [5]. Infection may also present clinically as a recurrent urinary tract infection (UTI) unresponsive to conventional antibiotics with one study in Russia finding a 25.8% rate of GUTB in patients with recurrent UTI [6]. As this was the initial presenting complaint of our patient, our case report highlights the value of considering GUTB amongst the differential diagnoses in such presentations.

European Association of Urology (EAU) guidelines recognize the challenge of diagnosing UGTB as there is no single diagnostic test. They recommend diagnosis guided by clinical suspicion based on the patient’s history and investigations. These investigations include smear microscopy, urine culture and nucleic acid amplification tests. Ultrasound and CT are the recommended imaging modalities however guidelines permit the use of MRI if CT is contraindicated, albeit conferring a low sensitivity [7].

First line treatment of UGTB is based on the WHO recommendation of a 6-month course of rifampicin, isoniazid, pyrazinamide, and ethambutol [8]. The EAU do not offer any recommendation for surgical management of GUTB due to paucity in high quality evidence in this area. It is suggested however that this is left to the discretion of the individual surgeon [7].

If detected early, GUTB has an excellent prognosis. Provided good adherence to anti-tubercular therapy, cure rate is around 90% [9]. However, failure to achieve this can result in considerable morbidity with complications including chronic renal failure, infertility and ureteric rupture further highlighting the importance of awareness for clinicians [2, 10].
